# Modified Resistance Water Therapy for Children With 22q11.2 Deletion Syndrome and Cleft Palate: A Pilot Observational Study

**DOI:** 10.1002/hsr2.73293

**Published:** 2026-10-08

**Authors:** Václav Baslík, Jitka Vokurková, Darina Čurdová, Petr Hájek, Radim Veselský, Petr Marcián, Olga Košková

**Affiliations:** ^1^ Cleft Center of the University Hospital Brno, University Hospital Brno Brno Czech Republic; ^2^ Department of Pediatric Surgery, Orthopedics and Traumatology University Hospital Brno Brno Czech Republic; ^3^ Department of Public Health, Faculty of Medicine Masaryk University Brno Czech Republic; ^4^ Department of Burns and Plastic Surgery University Hospital Brno Brno Czech Republic; ^5^ Faculty of Medicine Masaryk University Brno Czech Republic; ^6^ Institute of Solid Mechanics, Mechatronics and Biomechanics, Faculty of Mechanical Engineering Brno University of Technology Brno Czech Republic; ^7^ Institute of Manufacturing Technology, Faculty of Mechanical Engineering Brno University of Technology Brno Czech Republic

## Abstract

**Background and Aims:**

22q11.2 deletion syndrome (22q11.2DS) is frequently associated with cleft palate and persistent velopharyngeal dysfunction, resulting in hypernasality and reduced speech intelligibility despite surgical repair. Conventional therapies often provide inconsistent outcomes. This pilot study aimed to evaluate the feasibility and potential effects of modified resistance water therapy (MRWT) on speech outcomes in children with 22q11.2DS.

**Methods:**

This retrospective observational pilot study included nine children (aged 3–9 years) with genetically confirmed 22q11.2DS and a history of cleft palate (including submucous cleft palate) with persistent hypernasality after palatoplasty. MRWT was administered as a home‐based therapy using a silicone tube submerged in water, under regular supervision during follow‐up visits with a speech therapist at a cleft center. Speech was assessed using blinded ratings of hypernasality (4‐point scale), speech intelligibility (5‐point scale), and articulation (percentage of phonemes correct, PPC). Statistical analysis was performed using the Wilcoxon signed‐rank test, and inter‐rater reliability was assessed using Cohen's kappa.

**Results:**

Significant improvements were observed across all assessed speech parameters. Hypernasality decreased significantly, with median scores improving from 2 to 1 (*p* = 0.003), while speech intelligibility showed a similar improvement from 2 to 1 (*p* = 0.011). PPC increased from 70.5% to 92% (*p* = 0.004). Inter‐rater reliability was substantial to almost perfect (*κ* ≈ 0.79–0.89).

**Conclusion:**

MRWT appears to be a feasible, low‐cost, and non‐invasive adjunctive therapy with promising potential to improve speech outcomes in children with 22q11.2DS. Given the multifactorial etiology of velopharyngeal dysfunction in this population, MRWT may represent a useful addition to multidisciplinary care. However, larger prospective controlled studies are required to confirm its efficacy.

## 1 Introduction

22q11.2 deletion syndrome (22q11.2DS), also known as DiGeorge or velocardiofacial syndrome, is a genetic disorder associated with cleft palate or submucous cleft palate, often leading to velopharyngeal dysfunction (VPD) and hypernasality [1, 2, 3]. Despite palatoplasty, VPD can persist due to muscle hypotonia [4]. Conventional therapies like continuous positive airway pressure therapy or digital massage of soft palate often yield inconsistent outcomes [5, 6].

Modified resistance water therapy (MRWT), an adaptation of water resistance therapy (WRT) [7], offers a non‐invasive alternative aimed at stimulating soft palate musculature by periodic vibrations of the air column inside arise due to airflow resistance [8, 9, 10]. Historically, resonance‐based therapy dates to the 19th century with Gustav Spiess [11], followed by Sovijärvi in the 1950s, who introduced water‐submerged tubes [12, 13]. In recent decades, techniques like LAX VOX and DoctorVOX emerged [14], but few have targeted syndromic populations like 22q11.2DS. This therapy has been adapted for pediatric patients with 22q11.2DS at the authors' institution. The aim of this pilot observational study was to explore the potential effects and feasibility of MRWT in reducing hypernasality and improving speech intelligibility in children with 22q11.2DS.

## 2 Methods

### 2.1 Study Design and Participants

The study was approved by the Institutional Ethics Committee (Approval number: 06‐091024/EK). This retrospective observational pilot study was conducted at a tertiary referral center for cleft care. Patients were identified through institutional medical records and were consecutively recruited from those undergoing follow‐up for persistent speech difficulties after cleft palate repair between 2020 and 2023. Inclusion criteria were: genetically confirmed 22q11.2 DS, history of cleft palate or submucous cleft palate with prior palatoplasty at least 1 year before starting of MRWT, persistent hypernasality confirmed by a speech‐language therapist, and ability to comply with the MRWT protocol. Exclusion criteria included significant cognitive or behavioral impairment preventing cooperation, severe comorbidities affecting speech production, palatoplasty less than 1 year before starting MRWT, and incomplete follow‐up data. Total of nine patients met the inclusion criteria. All participants had undergone palatoplasty at least 12 months prior to initiation of MRWT to minimize the influence of surgery. During the study period, participants continued to receive standard speech therapy as part of routine clinical care.

### 2.2 MRWT Protocol

MRWT involved 5‐min home‐based daily exercises using a silicone tube submerged in water (Figure 1). The therapy was divided into two phases:

- Phase 1 (2–4 months): Non‐phonatory blowing into the tube, gradually increasing immersion depth to raise resistance (Supporting Information Videos 1 and 2).
- Phase 2 (3–12 months): Phonation ([u:]) through the tube to induce soft palate vibration (Supporting Information Video 3).

**FIGURE 1:**
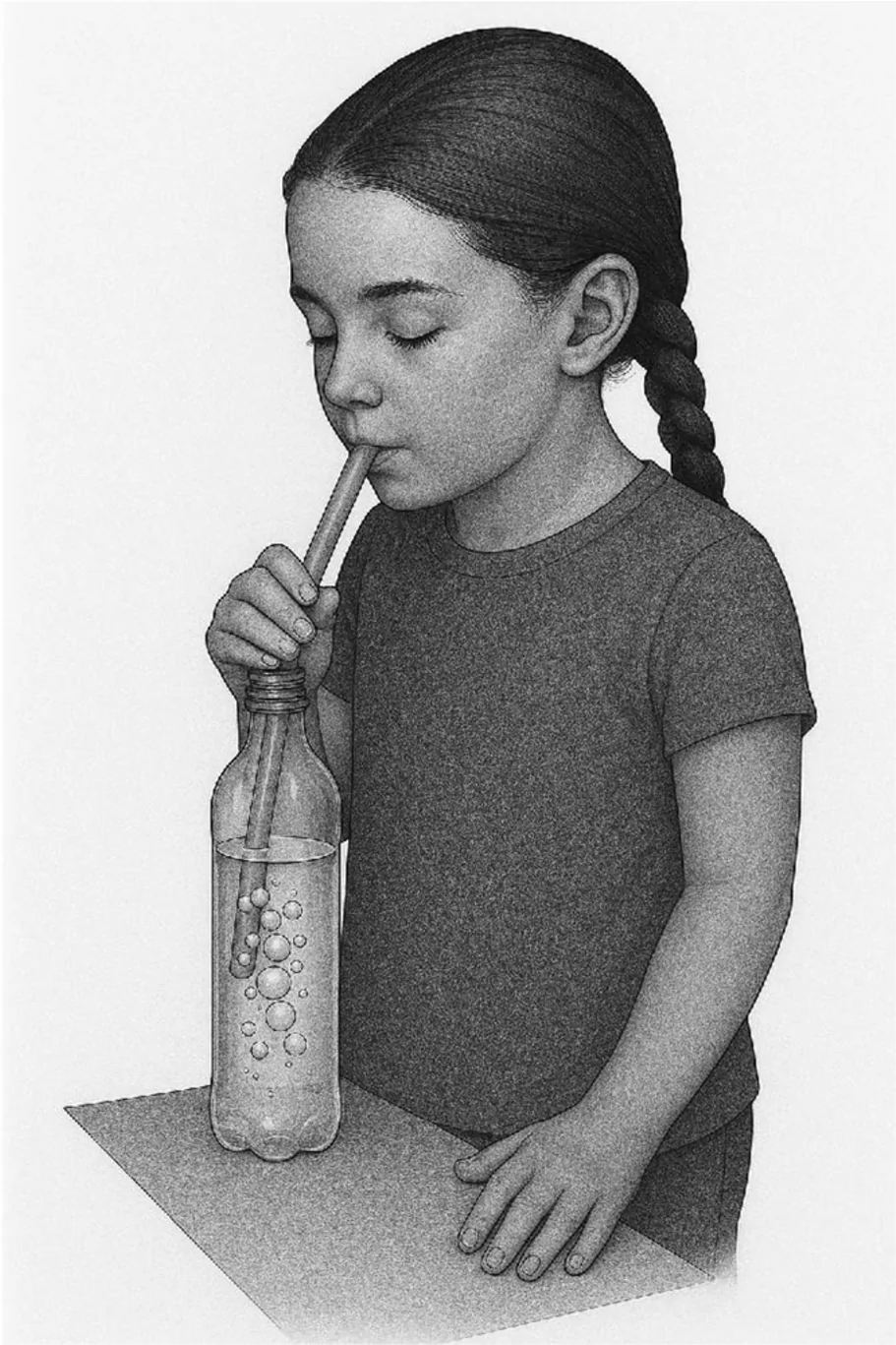
Demonstration of performing MRWT with the correct posture of the child (generated using ChatGPT‐5 (OpenAI, 2025) and reviewed, verified, and approved by the authors, who take full responsibility for the accuracy, interpretation, and final presentation of the AI‐generated content): A child exhales or phonates through a 30 cm silicone tube (12.5 mm inner diameter) submerged in a 1.5‐L plastic bottle filled with 1 L of room‐temperature water. The bottle is placed on a flat surface, and the tube is secured with an elastic band to prevent slipping. The child stands upright with relaxed shoulders during the exercise. The immersion depth of the tube was progressively increased from approximately 2 to 6–10 cm below the water surface (Phase 1). During Phase 2, children were instructed to produce a sustained vowel [u:] into the tube while maintaining continuous bubbling in the water. The immersion depth at which effective vibratory stimulation was achieved was individualized for each patient. Initiation of Phase 2 required prior mastery of Phase 1 at the maximum achievable immersion depth, which was individualized for each patient. Therapy duration ranged from 6 to 12 months, with bimonthly check‐ins by a speech‐language therapist. Adherence to the home‐based therapy was monitored through parental reports during these visits, during which the therapist also indicated progression between the two phases of therapy. Therapy was terminated upon minimization of hypernasality or upon reaching the maximal therapeutic effect, as evaluated after a maximum of 1 year of therapy.

### 2.3 Speech Assessment

Speech was evaluated using a structured Czech‐language protocol involving tasks such as picture naming and sentence repetition [16, 17]. Blinded raters (two speech‐language therapists—V.B. and D.Č.) assessed hypernasality on a 4‐point scale, speech intelligibility on a 5‐point scale, and articulation using the Percentage of Phonemes Correct (PPC) [15, 18]. The recordings of speech were anonymized and presented in randomized order.

### 2.4 Statistical Analysis

To assess the statistical significance of differences in hypernasality, speech intelligibility, and PPC scores before and after MRWT, the Wilcoxon signed‐rank test was used. A *p*‐value of less than 0.05 was considered statistically significant. Inter‐rater reliability was assessed using Cohen's kappa coefficient. Statistical analyses were performed using R (version 4.5.0; R Core Team, 2025).

## 3 Results

Nine children (aged 3–9) with genetically confirmed 22q11.2DS and persistent hypernasality after cleft palate surgery were included in the study. Cohen's kappa indicated substantial inter‐rater agreement for speech intelligibility (*κ* ≈ 0.79) and almost perfect agreement for hypernasality and PPC (*κ* ≈ 0.89). Table 1 summarizes the results of hypernasality, speech intelligibility, and articulation assessed before and after MRWT application. A statistically significant reduction was revealed in hypernasality, with a median score of 2 (pre‐MRWT, *n* = 9) decreasing to 1 (post‐MRWT, *n* = 9; *Z* = −2.93, *p* = 0.003, *r* = −0.98). Similarly, speech intelligibility showed a statistically significant improvement following the intervention, as evidenced by a change from a median of 2 (pre‐MRWT, *n* = 9) to 1 (post‐MRWT, *n* = 9; *Z* = −2.55, *p* = 0.011, *r* = −0.96). Articulation, quantified by PPC, also demonstrated an increase, moving from a median of 70.5% (pre‐MRWT, *n* = 9) to 92% (post‐MRWT, *n* = 9; W+ = 45, *p* = 0.004, *r* = 0.96). Effect sizes (*r* values) in all cases approached +/−1.0. Notably, all participants achieved individual gains in hypernasality (a 1‐point improvement in every case) and regarding speech intelligibility, seven out of nine participants (78%) experienced a 1‐point improvement.

**TABLE 1:** Characteristics of the 22q11.2DS cohort by age, sex, and cleft type and pre‐therapy and post‐therapy results in hypernasality, speech intelligibility, and articulation (PPC). Hypernasality: 1 normal, 2 mild, 3 moderate, 4 severe. Speech intelligibility: A score of 1 (Normal intelligibility) indicated speech that was completely intelligible, requiring no effort to understand. 2 (Mild impairment) was used when speech was mostly intelligible, though occasional misunderstandings occurred. 3 (Moderate impairment) described speech that was partly intelligible, with familiar content understood only with effort. 4 (Severe impairment) indicated that speech was mostly unintelligible, requiring frequent repetition or guessing based on context. Finally, 5 (Unintelligible) was given when the speaker's speech was unintelligible and the listener could not understand more than a few isolated words.

Patient no.	Age (years)	Sex	Cleft type	Hypernasality (pre‐MRWT)	Hypernasality (post‐MRWT)	Speech intelligibility (pre‐MRWT)	Speech intelligibility (post‐MRWT)	PPC% (pre‐MRWT)	PPC % (post‐MRWT)
1	6	F	Isolated cleft palate	2	1	1	1	75	96
2	8	M	Isolated cleft palate	2	1	1	1	72	92.7
3	5	M	Isolated cleft palate	3	2	2	1	52.7	80.7
4	4	F	Isolated cleft palate	3	2	2	1	70.5	90.5
5	7	M	Isolated cleft palate	2	1	2	1	46.5	68
6	3	M	Isolated cleft palate	2	1	2	1	64.4	92
7	9	F	Isolated cleft palate	3	2	3	2	62.9	84
8	5	F	Isolated cleft palate	2	1	2	1	81.8	95.3
9	6	F	Submucous cleft palate	2	1	2	1	88.7	98.2

## 4 Discussion

This preliminary observational pilot study suggests that MRWT may be associated with reductions in hypernasality and improvement in intelligibility and articulation in children with 22q11.2DS—a population known for complex velopharyngeal challenges [2, 19]. These children often exhibit persistent VPD post‐surgery due to both structural deficits and neuromuscular hypotonia [20], presenting unique therapeutic challenges.

MRWT offers a functional, non‐invasive approach that enhances soft palate coordination through vibratory stimulation [21]. Unlike traditional voice therapy tools, this protocol was specifically adapted for young children, integrating play and simplicity to boost compliance. WRT, originally developed for treating voice disorders [8, 9], has been adapted into MRWT for children with 22q11.2DS in authors institution. WRT is widely used among voice professionals, as it reduces phonatory effort, helps clear mucus from the vocal tract, and may relax laryngeal and pharyngeal muscles. It uses a silicone tube submerged in water, which creates airflow resistance and generates vibrations of the air column inside the vocal tract (Simberg et al.,2007 [7]). These vibrations resonate with the natural frequencies of vocal tract cavities and nearby tissues. The basic tuning of this resonance depends on the tube's length (typically 24–35 cm) and diameter (8–12 mm), while fine‐tuning is achieved by adjusting the immersion depth. MRWT builds on these principles but targets not only the vocal folds, as in traditional voice therapy, but also stimulates the soft palate and surrounding musculature. This resonance‐based stimulation may promote muscle toning and improved velopharyngeal closure. In our application for 22q11.2DS, tube dimensions were chosen to ensure optimal resonance within the pediatric vocal tract. MRWT thus provides a biomechanically grounded, non‐invasive method that complements surgical and conventional speech therapy approaches.

Despite promising results, limitations include the small sample size and absence of a control group. Improvements observed in this study may also be influenced by alternative factors, including natural speech development over time, variability in treatment duration, and the potential effects of concurrent clinical care, which could not be fully controlled due to the study design. Further multicenter trials are needed to substantiate these findings.

## 5 Conclusion

MRWT appears to be a feasible and low‐cost intervention with promising potential as a home‐based adjunctive therapy for children with 22q11.2DS. Its ease of application suggests that it may represent a useful adjunct within cleft care. Given the complex etiology of VPD in this population, MRWT holds promise as a targeted, non‐invasive therapeutic approach. However, further prospective controlled studies are needed to establish its effectiveness.

## Author Contributions

**Václav Baslík:** conceptualization, data curation, methodology, writing – original draft. **Jitka Vokurková:** data curation. **Darina Čurdová:** writing – original draft, investigation. **Petr Hájek:** investigation, writing – review and editing, methodology. **Radim Veselský:** writing – review and editing. **Petr Marcián:** validation, formal analysis, writing – review and editing, visualization. **Olga Košková:** conceptualization, project administration, supervision, methodology, visualization, writing – review and editing.

## Funding

The authors have nothing to report.

## Disclosure

Vaclav Baslik affirms that this manuscript is an honest, accurate, and transparent account of the study being reported; that no important aspects of the study have been omitted; and that any discrepancies from the study as planned (and, if relevant, registered) have been explained.

## Ethics Statement

The study was approved by the Ethics Committee of the University Hospital Brno (Approval number: 06‐091024/EK). Written informed consent of the participants was not needed due to the observational design of the study based on authorization by the Ethics Committee of the University Hospital Brno. The study was conducted in compliance with the principles of the Declaration of Helsinki.

## Conflicts of Interest

The authors declare no conflicts of interest.

## Policy on Using ChatGPT and Similar AI Tools

During the preparation of this manuscript, the authors used ChatGPT to improve grammar, flow, and readability. After using this tool, the authors reviewed and edited the content as needed and take full responsibility for the final version of the manuscript. ChatGPT‐5 (OpenAI, 2025) was also used to generate Figure 1. The authors reviewed, verified, and approved the AI‐generated figure and take full responsibility for its accuracy, interpretation, and final presentation.

## Supporting information


Supporting File 1

Supporting File 2

Supporting File 3

Supporting File 4

## Data Availability

The data that support the findings of this study are available from the corresponding author upon reasonable request.
