# Supplementary material for: Modified Resistance Water Therapy for Children With 22q11.2 Deletion Syndrome and Cleft Palate: A Pilot Observational Study
Source: Health Sci Rep. 2026 Oct 8;9(10):e73293. doi: 10.1002/hsr2.73293 (PMC13650404; doi:10.1002/hsr2.73293)
Supplement: Supplementary file 1 [file HSR2-9-e73293-s001.docx]

Video 1 Nasopharyngoscopic image of MRWT Phase 1 (minimum tube immersion).

Video 2 Nasopharyngoscopic image of MRWT Phase 1 (maximum tube immersion).

Video 3 Nasopharyngoscopic image of MRWT Phase 2 (with phonation of vowel [u:]).
